# Endogenous AhR agonist FICZ accumulates in rainbow trout (*Oncorhynchus mykiss*) alevins exposed to a mixture of two PAHs, retene and fluoranthene

**DOI:** 10.1007/s10646-022-02593-9

**Published:** 2022-10-11

**Authors:** Andreas N. M. Eriksson, Cyril Rigaud, Emma Wincent, Hannu Pakkanen, Pihla Salonen, Eeva-Riikka Vehniäinen

**Affiliations:** 1 Department of Biological and Environmental Sciences, University of Jyväskylä, Jyväskylä, Finland; 2 Institute of Environmental Medicine, Karolinska Institutet, Stockholm, Sweden; 3 Department of Chemistry, University of Jyväskylä, Jyväskylä, Finland

**Keywords:** Synergism, Mixture, PAH, FICZ, *Oncorhynchus mykiss*, Blue sac disease

## Abstract

Multiple studies have reported synergized toxicity of PAH mixtures in developing fish larvae relative to the additive effect of the components. From a toxicological perspective, multiple mechanisms are known to contribute to synergism, such as altered toxicodynamics and kinetics, as well as increased oxidative stress. An understudied contributor to synergism is the accumulation of endogenous metabolites, for example: the aryl hydrocarbon receptor 2 (AhR2) agonist and tryptophan metabolite 6-Formylindolo(3,2-b)carbazole (FICZ). Fish larvae exposed to FICZ, alongside knock-down of cytochrome p450 (*cyp1a*), has been reported to induced symptoms of toxicity similar to those observed following exposure to PAHs or the dioxin 2,3,7,8-tetrachlorodibenzo-*p*-dioxin. Here, we explored if FICZ accumulates in newly hatched rainbow trout alevins (*Oncorhynchus mykiss*) exposed to two PAHs with different properties: retene (potent AhR2 agonist) and fluoranthene (weak AhR2 agonist and Cyp1a inhibitor), either alone or as a binary mixture for 3 and 7 days. We found that exposure to the mixture resulted in accumulation of endogenous FICZ, synergized the blue sac disease index (BSD), and altered the body burden profiles of the PAHs, when compared to the alevins exposed to the individual components. It is thus very plausible that accumulation of endogenously derived FICZ contributed to the synergized BSD index and toxicity in exposed alevins. Accumulation of endogenously derived FICZ is a novel finding that extends our general understanding on PAHs toxicity in developing fish larvae, while at the same time highlighting why environmental risk assessment of PAHs should not be based solely results from the assessment of individual compounds.

## Introduction

Polycyclic aromatic hydrocarbons (PAHs) are a diverse and widespread group of environmental pollutants of either natural or anthropogenic origin (Wickström and Tolonen 1987; dos Santos et al. 2018) and are always present as complex mixtures (Tissot and Welte 1978). From an aquatic toxicological perspective, the exact mechanisms of toxicity are not fully understood, even after decades of research employing multiple species of fish and types of PAHs (Heintz et al. 2000; Brinkworth et al. 2003; Wassenberg and Di Giulio 2004; Van Tiem and Di Giulio 2011; Clark et al. 2013; Rigaud et al. 2020a). What is known is that different PAHs induce toxicity through different mechanisms and cause exposure and PAH specific toxicity profiles (Billiard et al. 2008; Incardona et al. 2011; Geier et al. 2018; Rigaud et al. 2020a, b; Eriksson et al. 2022a, b). Additionally, heart structure and function are especially sensitive to the influence of PAH(s) during early life development of fish (Incardona et al. 2011, 2009; Vehniäinen et al. 2016). The specificity of cardiotoxicity is hypothesized to be linked to interaction(s) between PAHs, as a part of crude oil, and lipoproteins bound to the cellular membrane (Incardona 2017). Furthermore, mixture composition has been observed and reported to modulate PAH toxicity outcome, relative to the components (Geier et al. 2018; Eriksson et al. 2022a, b). The best-known molecular mechanism related to PAH toxicity is that involving activation of the aryl-hydrocarbon receptor 2 (AhR2) (Massarsky et al. 2016; Doering et al. 2019). Activation of AhR2, by PAHs such as retene (Scott et al. 2011), benzo[a]pyrene (Incardona et al. 2011; Song et al. 2019), benzo[k]fluoranthene (Van Tiem and Di Giulio 2011), and pyrene (Incardona et al. 2011) induces the expression of, among many genes, cytochrome P450 (CYP1), particularly *cyp1a*, which encodes for an enzyme that initiates phase I metabolism of xenobiotics through hydroxylation (Billiard et al. 2008). By contrast, some PAHs, such as fluoranthene, can both induce the expression of *cyp1a*, and inhibit the function of the translated and functional enzyme by blocking its active site (Willett et al. 1998), which has previously been shown to synergize the toxicity of other PAHs (Wills et al. 2009; Van Tiem and Di Giulio 2011).

A commonly assessed biomarker of PAH toxicity in developing fish is the so called blue sac disease syndrome (BSD), which encompasses several symptoms of PAH induced toxicity: craniofacial deformities, hemorrhaging, yolk and pericardial edema and spinal curvatures (Billiard et al. 1999, 2006; Colavecchia et al. 2006). Although it is not fully known how BSD is induced (Scott et al. 2011; Clark et al. 2013), knockdown of *ahr2* (but not *cyp1a*) is known to prevent the formation of BSD-related symptoms in developing zebrafish larvae (*Danio rerio*), which implies that downstream molecular events of AhR2, other than activation of Cyp1, are required for the manifestation of PAH toxicity (Van Tiem and Di Giulio 2011; Massarsky et al. 2016). Recently, the activation of cyclooxygenase-2 (*cox2*), which is linked to AhR2 activation, by PAH(s) has been reported to be involved in the induction of developmental toxicity in fish (Doering et al. 2019).

Some endogenous compounds, such as the tryptophan derivative FICZ (6-Formylindolo[3,2-b]carbazole), are also known to be able to activate AhR2 (Wincent et al. 2009). FICZ is a very potent AhR agonist, and during normal conditions, it is maintained at low levels by constant Cyp1-mediated metabolism (Wincent et al. 2009). Increased rate of formation of FICZ has been observed following three distinct processes: increased enzymatic activity, increased UV-irradiation, and increased levels of oxidative stress (Smirnova et al. 2016; Rannug and Rannug 2018). Newly hatched zebrafish larvae exposed to externally administrated FICZ, in combination with a Cyp1a-inhibitor (alpha-naphthoflavone) or *cyp1a*-knockdown, resulted in reduced metabolism of FICZ and increased occurrence of BSD-related symptoms, and other signs of toxicity that resemble those caused by exposure to PAHs (Wincent et al. 2016). It has therefore been hypothesized that the formation and accumulation of endogenous FICZ could contribute to AhR-mediated developmental toxicity in fish larvae (Wincent et al. 2012; Rannug and Rannug 2018).

Therefore, we investigated if exposure to two PAHs with different modes of action, retene (an AhR2 agonist) and fluoranthene (a weaker AhR2 agonist and Cyp1a inhibitor) (Barron et al. 2004), alone or as a binary mixture, could force the accumulation of endogenously derived FICZ in newly hatched and developing rainbow trout alevins (*Oncorhynchus mykiss*). By assessing the temporal development of the PAH and FICZ specific body burdens, in relation to the BSD index in developing rainbow trout, we aimed at uncovering how potential accumulation of FICZ could contribute to developmental toxicity.

## Materials and methods

### Experimental setup

Newly hatched 360 degree-days and healthy rainbow trout alevins (free from developmental deformities, edemas, and hemorrhages; provided by Hanka-Taimen Oy fish farm, Central Finland) were either exposed to dimethyl sulfoxide as control (DMSO; 20 µl L^−1^; Sigma–Aldrich, St-Louis, MO, USA) CAS-number 67-68-5), retene (nominally 32 µg L^−1^; MP Biomedicals, Illkirch, France) CAS-number 483-65-3), fluoranthene (nominally 50 µg L^−1^; Sigma Aldrich; CAS-number 206-44-0) or the binary mixture of the two PAHs (at aforementioned nominal concentrations), and sampled after 1, 3 and 7 days of exposure. Investigated nominal PAH concentrations were selected as to provoke toxicity, but not increase mortality rates, as per previously published studies on retene toxicity (Hodson et al. 2007; Scott and Hodson, 2008; Vehniäinen et al. 2016) and an unpublished assessment of fluoranthene toxicity (rainbow trout alevin exposed to 500 µg L^−1^ for 11 days, did not increase mortality). Additionally, the concentration of DMSO (20 µl L^−1^) can be considered as acceptable (<100 µl L^−1^; OECD 2013), and should not contribute to solvent-mediated toxicity (Maes et al. 2012). Exposures were performed in 1.5 litre Pyrex glass bowls, filled with 1 litre of filtered lake water obtained from Konnevesi Research station in Central Finland. Each treatment was performed in triplicates, and each replicate contained 15 alevins. Exposure water temperature was maintained at 10.8 ± 0.3 °C, and a 16:8 light to dark ratio employed. Relative oxygen saturation, pH and conductivity were measured at 103.23 ± 3.32%, 7.38 ± 0.09 and 17.53 ± 3.95 mS m^−1^, respectively and throughout the exposure duration. At sampling, 5 alevins per replicate were video recorded and photographed while the remaining 10 alevins (per replicate) were photographed and snap-frozen for later HPLC-analysis. Symptoms of blue sac disease (BSD) were assessed based upon the video recordings (pericardial edema) combined with photographs (yolk sac edema and hemorrhages) of the 5 individually sampled alevins in silico.

### Blue sac disease index

BSD index was calculated through established convention for each exposure replicate (*n* = 3; Eq. 1) (Colavecchia et al. 2006; Scott et al. 2011). In order to compare the BSD indices reported by Eriksson et al. (2022a) with those obtained in this present study, the same symptoms of toxicity were assessed and scored in the same fashion: hemorrhages (HE; scored 0 or 1; not present or present), pericardial (PE; 0 or 1) and yolk sac edemas (YE: 0 or 1). The total score per replicate was then divided by the total maximum potential score per replicate: 15 (maximum score per individual alevin was 3, and 5 alevins per replicate were assessed).

1 $$BSD\,index = \frac{{\mathop {\sum }\nolimits HE + \mathop {\sum }\nolimits PE + \mathop {\sum }\nolimits YE}}{{15}}$$

### Establishment of body burden through HPLC analysis and confirmation of FICZ

Preparation of alevins and HPLC analysis were performed as instructed by Rigaud et al. (2020a) and Eriksson et al. (2022a). In short, the 10 pooled alevins (per replicate) were homogenized by zirconium pellets (circumference of 1 and 2 mm; Next Advance, USA) in 70% acetonitrile (ACN; Fisher Scientific) using a standard model bullet blender (Next Advance). The homogenate was then centrifuged (Centrifuge 5415 R, Eppendorf, Germany) for 10 min at 14,000 rpm and 4 °C. The supernatant was collected and the pellet re-suspended in 70% ACN, centrifuged, the supernatant collected and pooled. The re-suspension step was performed twice. Body burden of the PAHs and FICZ was established through HPLC with fluorescence detector (Shimadzu UHPLC Nexera-system). Detection parameters and limits of the HPLC measurements are presented in Table 1. Subsequent area under the curve (AUC) for each respective compound was manually adjusted, background compensated (control AUC), and the body burden calculated based upon standard curves.

**Table 1:** PAH and FICZ detection parameters employed for Shimadzu HPLC analyses

HPLC and quantification parameters	Retene	Fluoranthene	FICZ
Excitation (nm)	259	288	390
Emission (nm)	370	525	525
Retention time (minutes)	16.7 ± 0.1	13.5 ± 0.2	8.370 ± 0.001
LOD (nM)	4.56	6.21	15.11
LOQ (nM)	13.83	18.83	45.79

The presence of FICZ was confirmed using LC-MS/MS (Agilent 1290 ultra-high-pressure liquid chromatography system coupled to an Agilent 6460 triple quadrupole mass spectrometer). The mobile phase employed in LC-MS/MS analysis was created from double-distilled water and acetonitrile (70%), both fortified with 1.5 mM formic acid (Fischer Scientific). The ratio between the components of the mobile phase started at 1:1 which from minute 1 to 9 increased to 1:20 before returning to 1:1 from minute 10.5 to 11; finally, the post-time column stabilization lasted for 2.5 min. Retention time for FICZ was 3.54 min (LC-MS/MS).

### qPCR preparation and analysis

Measurement of whole-body *cyp1a* expression was performed according to instructions reported by Rigaud et al. (2020a), which in turn was modified from Sivula et al. (2018). In short: the 5 individual alevin carcasses were treated using TRI reagent (Molecular Research Centre) in order to extract RNA. The concentration, purity (NanoDrop 1000, Thermo Fisher Scientific) and integrity of the RNA were assessed using a TapeStation and confirmed (eukaryote total RNA 6000 Nano-kit; Agilent) in accordance with the manufacturer’s instructions. RNA was DNase treated (DNase I, Fermentas) and using iScript cDNA Synthesis Kit (Bio-Rad, USA), 500 ng of RNA was reverse transcribed into cDNA and diluted 1:10 in nuclease-free water. Five µL of diluted cDNA were then mixed with 1.5 µL of forward and reverse primers (final concentration 300 nM; Table 2), 4.5 µL sterile H_2_O and 12.5 µL of iQ SYBR Green Supermix (Bio-Rad). The 25 µL qPCR reaction mixture was analyzed utilizing the CFX96 Real-Time PCR cycler (Bio-Rad) according to established protocol: 3 min at 95 °C; 40 cycles (10 s at 95 °C, 10 s at 58 °C, 30 s at 72 °C, 10 s at 95 °C) and melting curve increased from 55 °C to 95 °C in increments of 0.5 °C. The expression of *cyp1a* was calculated using Bio-Rad CFX Manager software (v.3.1), employing *ndufa* and *rl17* as references genes (Table 2).

**Table 2:** Accession identification codes, forward and reverse primer sequences (F = forward; R = reverse), subsequent product length (base pairs) and overall efficiency for *cyp1a* (%) and the reference genes (*ndufa* and *rl17*)

Gene	Accession	Primer	Product length	Efficiency (%)
*cyp1a*	NM_001140880.2	F: CAGTCCGCCAGGCTCTTATCAAGC	94	96.9
		R: GCCAAGCTCTTGCCGTCGTTGAT		
*ndufa*	NM_001195159.2	F: ATCGAGCACATCCAGGTAACAAG	99	110.1
		R: AATGTGGCAAGGGGAGCTCATGTA		
*rl17*	NM_001160582.1	F: TTCAGAGCCTCATCTTGCCTGCT	119	114
		R: CAACATAGGGATTGGAGAGCTGTACG		

### Data analysis

Statistical analyses were performed using R v.4.0.3 (The R Foundation for Statistical Computing, 2020) coupled with R-studio v.1.3.1093 (RStudio Team 2020). Significant differences were assessed using Kruskal–Wallis test combined with Dunn’s *post-hoc* test (KW + Dunn) and adjusted for multiple comparisons using Bonferroni’s method (Benjamini and Hochberg 1995). Exposure specific changes in the body burden of mixture exposed alevins, compared to those exposed to the components, were assessed using Mann–Whitney’s *U*-test.

In order to assess the effect of the mixture, relative to the additive effect of the components on the obtained BSD indices, background adjusted combination indices were calculated (CI; Eq. 2) (Foucquier and Guedj 2015) and compared with the results reported by Eriksson et al. (2022a):

2 $$CI = \frac{{R + F - \left( {R \times F} \right)}}{M}$$

where the average, and normalized effect (against control), exerted by retene and fluoranthene alone are represented by R and F, while the normalized effect exerted by the binary mixture is represented by M. A stronger effect by the mixture, relative to the components, is assumed if the CI is <1.

## Results and discussion

The occurrence of blue sac disease related symptoms was dependent on both the type of exposure and duration (Table 3). Exposure to fluoranthene produced a weaker index after 3 days of exposure compared to Day 7. Exposure to retene produced, on average, similar BSD indices on both Day 3 and 7. By contrast, exposure to the binary mixture produced the strongest BSD index, irrespective of exposure duration. Yet, significance was only observed after 3 days of exposure (fluoranthene relative to mixture), while near-to-significance was obtained between control and mixture exposed alevins on Day 7 (*p* = 0.0532). Few replicates per treatment (*n* = 3) are plausibly obscuring some the statistical outcome, as the BSD indices reported by Eriksson et al. (2022a), who utilized a greater number of replicates (*n* = 6), were accompanied by statistical differences. Nevertheless, exposure to the mixture resulted in a greater than predicted BSD index (Table 3; irrespective of exposure duration), as the combination index was 0.39 after 3 days of exposure and 0.86 by Day 7. Hence, the results presented in Eriksson et al. (2022a) can therefore be considered as comparable, highlighting BSD indices as a functional proxy of nominal exposure concentrations in a standardized exposure study; at least when considering the effect of this binary mixture and combination indices.

**Table 3:** Average (± standard deviation) blue sac disease (BSD) indices among alevins exposed for 3 and 7 days to DMSO (control) and the PAHs retene (Ret) and fluoranthene (Flu) or the binary mixture

Study	Exposure	BSD; Day 3	BSD; Day 7	Background adjusted; Day 3;	Background adjusted; Day 7
Eriksson et al. (2022a)	DMSO	0.20 ± 0.08 ^a^	0.23 ± 0.11 ^A^	0	0
	Flu	0.34 ± 0.17 ^ab^	0.25 ± 0.06 ^A^	0.14	0.02
	Ret	0.25 ± 0.08 ^a^	0.29 ± 0.16 ^AB^	0.05	0.06
	Mix	0.49 ± 0.09 ^b^	0.43 ± 0.10 ^B^	0.29 ^#^	0.20 ^#^
Present study	DMSO	0.20 ± 0.12 ^12^	0.24 ± 0.08	0	0
	Flu	0.18 ± 0.10 ^1^	0.33 ± 0.12	−0.02	0.09
	Ret	0.33 ± 0.18 ^12^	0.33 ± 0.12	0.13	0.09
	Mix	0.49 ± 0.10 ^2^	0.44 ± 0.10 ¤	0.29 ^#^	0.20 ^#^

¤ DMSO – Mix: *p* = 0.0532 (Dunn’s *post hoc* test)

Significant differences, as per Eriksson et al. (2022a), are denoted with different lower and uppercase letters for Day 3 and 7, respectively (*N* = 6)

A near-to significant difference between control and mixture, by Day 7, are denoted with ¤ (Present study)

Significant differences (KW + Dunn; *N* = 3) were observed among mixture exposed alevins compared to control (DMSO) and fluoranthene by Day 3 (denoted with different numbers)

Background adjusted indices were utilized for the assessment of combination indices

A BSD-index greater than the combined additive effect of the components among alevins exposed to the mixture, as per combination index

BSD results, as per this present study, are compared with the indices reported by Eriksson et al. (2022a)

Even though the actual PAH concentrations in water were not measured, we know from previous exposure studies that the concentrations of PAH in newly made solutions are very similar to the nominal concentration (Honkanen et al. 2020). Moreover, and as presented in Table 3, the same nominal concentrations caused very similar BSD indices as in the present study, relative to our previous work (Eriksson et al. 2022a), whereby strengthening the comparability.

**Fig. 1:**
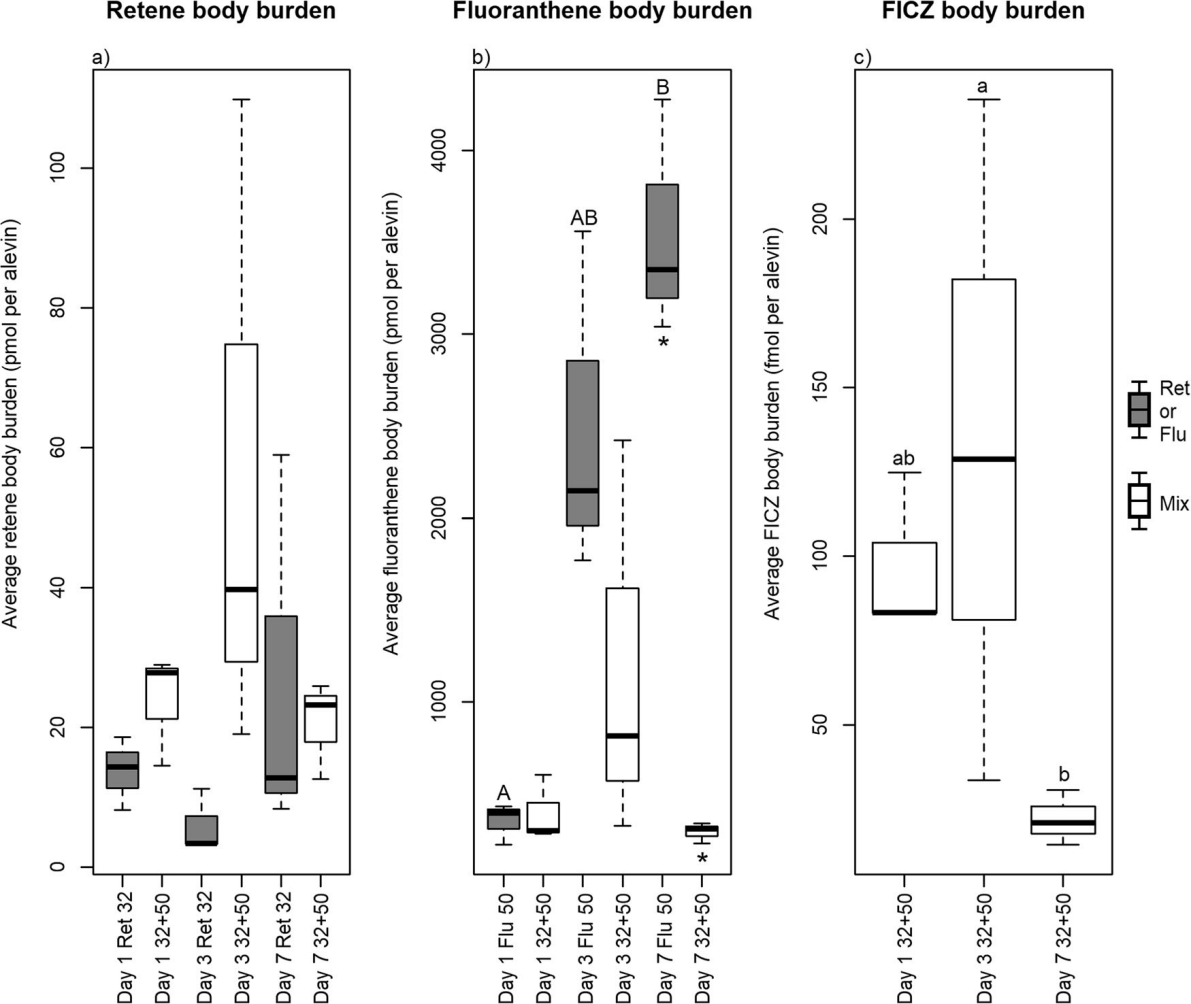
Boxplot representation of the exposure specific body burden profiles, per alevin, following exposure to retene (**a**; pmol), fluoranthene (**b**; pmol) and endogenously formed FICZ (**c**; fmol). Alevins were sampled after 1, 3 and 7 days of semi-static exposure to the PAHs alone (dark-grey-filled boxes) or as a binary mixture (white-filled boxes). Significant differences in body burden within each treatment, as per KW + Dunn, are denoted with upper- (fluoranthene) and lower-case letters (FICZ). Significant differences in body burden between larvae exposed to the mixture and the components are denoted with *. N per treatment = 3

The body burden of retene fluctuated non-significantly with time, irrespective of treatment (Fig. 1a). Hence, no significant difference in the body burden of retene was observed in mixture exposed alevins relative to those exposed to retene alone. By comparison, exposure to fluoranthene alone resulted in an increasing body burden with time, and a significantly greater body burden was observed by Day 7 compared to Day 1 (Fig. 1b). When co-exposed with retene, the body burden of fluoranthene diminished significantly compared to alevins exposed to fluoranthene alone for 7 days. Similar temporal patterns of accumulation were observed among alevins exposed to the mixture of retene and fluoranthene, as reported in our previous studies (Eriksson et al. 2022a, b). Hence, the reduction in the body burden of fluoranthene, when co-exposed with retene, reflects a broader and more potent activation of phase I and II metabolic processes, either transcriptomic (Eriksson et al. 2022a) or proteomic (Eriksson et al. 2022b), that can offset the inhibitory effect of fluoranthene upon Cyp1a.

Additionally, we were able to identify and quantify the accumulation of endogenously formed FICZ in alevins exposed to the mixture, but not in alevins exposed to fluoranthene or retene alone (Fig. 1c). However, it cannot be ruled out that exposure to retene and fluoranthene alone increased the rate of formation. Rather, it can only be stated that accumulation to detectable levels did not occur following exposure to the individual PAHs. Endogenously formed and accumulated FICZ can either be derived enzymatically from tryptamine or tryptophan, following UV-irradiation, or oxidation of tryptophan, as observed during increased oxidative stress (Smirnova et al. 2016; Rannug and Rannug 2018). UV-irradiation can be rejected as causative agent due to the architecture of the exposure facility (no windows), as the room was illuminated by yellow florescent light. That leaves enzymatic processes and oxidative stress as the most plausible causes; the latter being more likely due the known and established relationship between PAH toxicity and subsequently increased oxidative stress (Timme-Laragy et al. 2007; Song et al. 2019), altered iron metabolism (Rigaud et al. 2020b; Eriksson et al. 2022b) and activation of heat shock proteins (Räsänen et al. 2012) in PAH exposed fish larvae. The body burden of FICZ may also increase when subsequent metabolism by Cyp1a is inhibited (Wincent et al. 2012, 2016). In the present study, the body burden of FICZ peaked by Day 3 before decreasing significantly by Day 7. The dynamics of the body burden of FICZ, over time, thus suggests a link between actual Cyp1a inhibition and subsequent accumulation of FICZ in relation to development, and plausibly influenced by the maturation of the liver. However, Cyp1a inhibition by exposure to fluoranthene alone was not sufficient in causing accumulation of FICZ. Therefore, it can be postulated that alterations of multiple parallel molecular processes and events are required for FICZ accumulation in vivo.

Accumulation of PAHs and FICZ was reflected in the expression of *cyp1a* following 3 days of exposure. Exposure to fluoranthene resulted in a non-significantly increased expression relative to control, whereas exposure to retene increased the expression significantly. By contrast, exposure to the mixture resulted in a significantly stronger expression, relative to the other treatments and as a consequence, the measured expression was greater than the predicted additive effect exerted by the components (Table 4; combination index), results that were expected as per previous studies (Billiard et al. 2008; Eriksson et al. 2022a). As the experiment was designed to detect and quantify FICZ, it can only be assumed that accumulation of endogenously derived FICZ influences the expression of *cyp1a*. The underlying processes governing the toxicodynamic and kinetic processes are yet to be determined.

**Table 4:** Whole-body *cyp1a* expression (%) following 3 days of exposure to DMSO (control treatment), fluoranthene (Flu), retene (Ret) and the binary mixture (Mix)

Treatment	*cyp1a* expression (%; relative control)	Background adjusted *cyp1a expression*	*N*
DMSO	100 ± 53^1^	0	9
Flu	159 ± 64^12^	59	9
Ret	436 ± 253^23^	336	7
Mix	2278 ± 1488^3^	2178 ¤	7

Significant differences are denoted by different numbers (KW + Dunn), while a stronger, background adjusted, cyp1a expression among mixture exposed alevins is highlighted by ¤ (as per combination index)

Combined, these findings highlight that the toxicity exerted by this mixture of PAHs could not have been predicted from the additive effect of the components in developing rainbow trout alevins, nor could the observed synergized BSD index in alevins exposed to the mixture be explained by the PAHs body burden alone. As FICZ is known to cause symptoms of BSD in fish (Wincent et al. 2016), it is plausible that accumulation of FICZ could contribute to the synergized BSD index among mixture exposed alevins. This is a novel mechanism of PAH mixture toxicity that could, at least, partly explain the synergism observed in organisms exposed to complex PAH mixtures such as crude oil (Billiard et al. 2008). However, it is unknown to what extent accumulated FICZ contributes to toxicity, nor if accumulation can occur in situ following environmental contamination.

## Conclusions

Accumulation of endogenously derived FICZ is a novel discovery that will impact how PAH mixture toxicity is perceived. Accumulation of FICZ, which is a known AhR2 agonist that has been observed to induce developmental toxicity in zebrafish larvae, is likely to have influenced and aggravated developmental toxicity, as per the strong BSD indices. The underlying processes promoting accumulation are, in this case, unknown. Hypothetically, accumulation can be due to 1) decreased rate of xenobiotic metabolism due to increased substrate competition between the PAHs and FICZ for phase I and II metabolic enzymes; 2) increased rate of formation; or 3) a combination of decreased rate of phase I and II metabolism alongside increased rate of formation. Additionally, it is unknown if formation and accumulation of FICZ is tissue specific, evenly distributed throughout the developing organisms or produced in specific tissue(s) but distributed evenly. Moreover, it is unknown, although plausible, that exposure to other types of PAH mixtures (simple and complex), or crude oil, can result in the accumulation of FICZ. The same is true for species and life stage specificity, which must also be assessed. Therefore, more research on the nature of FICZ, in relation to developmental toxicity, toxicodynamics and kinetics are required to further the understanding of PAH toxicity in fish.
